# Dose-Response Effects of a Web-Based Physical Activity Program on Body Composition and Metabolic Health in Inactive Older Adults: Additional Analyses of a Randomized Controlled Trial

**DOI:** 10.2196/jmir.3643

**Published:** 2014-12-04

**Authors:** David P Vroege, Carolien A Wijsman, Karen Broekhuizen, Anton JM de Craen, Diana van Heemst, Frans JG van der Ouderaa, Willem van Mechelen, P Eline Slagboom, Michael Catt, Rudi GJ Westendorp, Evert ALM Verhagen, Simon P Mooijaart

**Affiliations:** ^1^Department of Gerontology and GeriatricsLeiden University Medical CenterLeidenNetherlands; ^2^Institute for Evidence-Based Medicine in Old Age | IEMOLeidenNetherlands; ^3^Leyden Academy on Vitality and AgeingLeidenNetherlands; ^4^Netherlands Consortium for Healthy AgingLeidenNetherlands; ^5^Department of Public & Occupational Health and EMGO+ InstituteVU University Medical CenterAmsterdamNetherlands; ^6^Department of Medical Statisticssection of Molecular EpidemiologyLeiden University Medical CenterLeidenNetherlands; ^7^Institute for Aging and HealthNewcastle UniversityNewcastle upon TyneUnited Kingdom

**Keywords:** Internet, physical activity, aging, metabolism

## Abstract

**Background:**

Low physical activity is a major risk factor for several age-related diseases. Recently, we showed in a randomized controlled trial that a 12-week Web-based intervention (Philips DirectLife) to increase physical activity was effective in increasing physical activity levels and metabolic health in an inactive population aged 60-70 years.

**Objective:**

The goal of this paper was to assess how many participants successfully reached the physical activity level as targeted by the intervention and what the effects of the intervention on body composition and metabolic health in these successful individuals were to provide insight in the maximum attainable effect of the intervention.

**Methods:**

Among the 235 participants in a randomized controlled trial of the Actief en Gezond Oud (AGO) study, we assessed the effects of the intervention on metabolic parameters in those who had successfully reached their personalized physical activity target compared with the entire intervention group. Furthermore, we studied the dose-response effect of increase in physical activity on metabolic outcome within the intervention group.

**Results:**

Of the intervention group, 50 of 119 (42.0%) participants successfully reached the physical activity target (corresponding to a 10% increased daily physical activity on average). This group showed markedly higher effects of the intervention compared to the entire intervention group, with greater decreases in body weight (2.74 vs 1.49 kg), waist circumference (3.74 vs 2.33 cm), insulin resistance (HOMA index: 0.23 vs 0.20), and in cholesterol/HDL ratio (0.39 vs 0.20) and Framingham risk score (0.90% vs 0.54%). We found that men compared to women were more likely to be successful. The dose-response analysis showed that there was a significant association between increase in minutes spent in moderate-to-vigorous activity and body weight loss, BMI reduction, waist circumference reduction, HDL cholesterol increasing, and cholesterol/HDL ratio lowering.

**Conclusions:**

Of the intervention group, 42.0% (50/119) reached their daily physical activity end goal, which was associated with a markedly better effect on body composition and metabolic health compared to the effect in the entire intervention group. In this population, men are more likely to be successful in increasing physical activity. Findings demonstrate that improving the effect of such physical activity interventions requires finding new ways to increase the proportion of the population reaching the targeted goal.

**Trial Registration:**

Dutch Trial Registry: NTR 3045; http://www.trialregister.nl/trialreg/admin/rctview.asp?TC=3045 (Archived by WebCite at http://www.webcitation.org/6KPw52dCc).

## Introduction

Insufficient physical activity is pandemic and a major risk factor for several lifestyle- and age-related conditions, including cardiovascular disease, diabetes mellitus, and cognitive decline [1-7]. Intervention studies directed at increasing physical activity in older people have shown to be effective in improving metabolic health in older populations [8,9]. However, most physical activity interventions have used face-to-face communication, making them costly and time-consuming, thus hampering the potential of implementation as preventive programs at a larger scale. There is a need for new and effective intervention strategies that allow for large-scale implementation.

Recently, we performed the Actief en Gezond Oud (AGO) study, a randomized controlled trial of the effect of a 3-month Web-based intervention program targeted at improving physical activity in inactive older adults [10]. The intervention program (Philips DirectLife) consisted of the use of an accelerometer, online feedback, and coaching over the Internet. Results showed that the intervention was effective in increasing objectively measured physical activity and in improving metabolic health in inactive older adults in the total study population. However, this intention-to-treat analysis did not include analyses of treatment success at the level of the individual. Understanding the proportion of the study population that successfully reached the physical activity target and the effects of the intervention in these individuals could contribute to gaining insight in the maximum attainable result of such interventions, allowing future interventions to optimize effectiveness by target specific populations or adjusting the target physical activity level [11,12].

In the present paper, we performed additional analyses on the AGO study data. First, we analyzed what proportion of participants successfully reached the physical activity target of the program. Second, we analyzed the effect on metabolic outcomes of the intervention in those participants who successfully reached the physical activity target. Third, we performed a dose-response analysis of increasing physical activity on metabolic outcomes among all participants in the intervention group.

## Methods

### Study Design and Participants

All analyses of this paper were performed with data obtained from a previously reported randomized controlled trial into the effects of a 3-month Web-based intervention program targeted at enhancing levels of daily physical activity in inactive older adults: The AGO study [10]. In short, this study recruited participants aged 60 to 70 years from the region of Leiden, The Netherlands, through advertisement in local newspapers and press notification, directing participants motivated to increase physical activity to the study website, where they completed an online questionnaire. Inclusion criteria were (1) age between 60 and 70 years and (2) possession of and knowledge on how to use a personal computer. Exclusion criteria were (1) active lifestyle as assessed by the General Practice Physical Activity Questionnaire (GPPAQ), (2) history of diabetes or use of glucose lowering medication, and (3) physical inability or medical contraindication to increase physical activity level. The presence of an inactive lifestyle was then assessed by a self-report physical activity questionnaire (GPPAQ) [13]. The GPPAQ asks questions about average physical activity over the past week of the participant and categorizes people into 4 levels of physical activity. We excluded participants in the highest level of physical activity, which corresponded to performing more than 3 hours of self-reported exercise and cycling combined weekly. At baseline, visit participants were randomly assigned to the intervention group or a waiting list control. Randomization was performed by a computerized program for intervention versus waiting list control in a ratio of 1:1 with a block size of 12. Stratification was performed by gender. Concealment of treatment allocation was ensured by randomizing at the end of the first study visit after all baseline measurements and instructions at the study center were completed. The study was approved by the medical ethical committee of Leiden University Medical Center, The Netherlands. An independent physician was available for questions regarding study information. This study was registered with the Dutch Trial Registry (NTR 3045).

### Intervention

**Figure 1:**
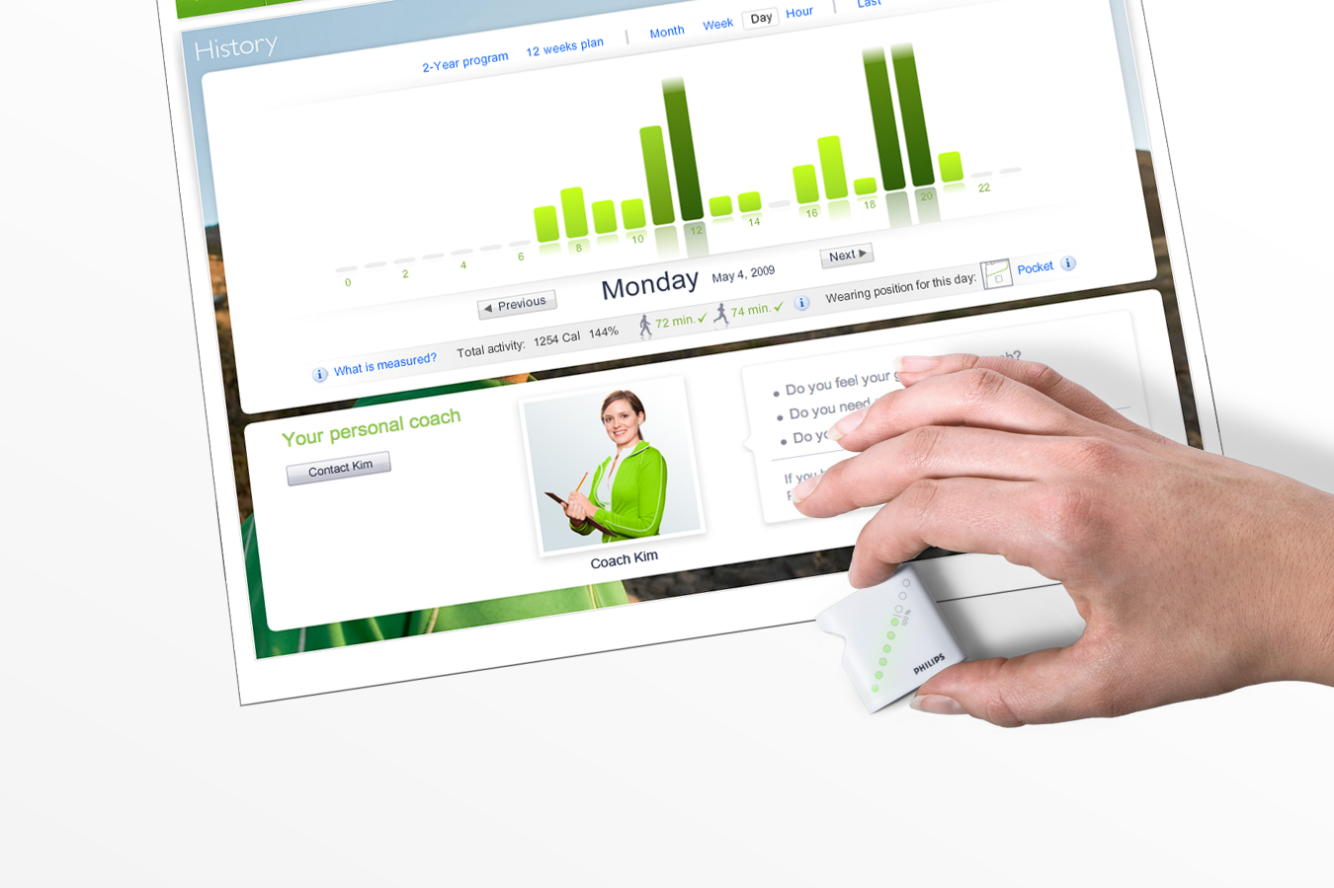
Screenshot of DirectLife intervention program and accelerometer.

Participants in the intervention group received a commercially available Web-based physical activity program (DirectLife, Philips, Consumer Lifestyle, Amsterdam) directed at increasing daily physical activity. The DirectLife program is based on established health behavior change models [14,15], and takes into account the individual’s current daily physical activity level and subsequently provides a personalized goal. Briefly, DirectLife consists of 3 elements: (1) an accelerometer-based physical activity monitor, (2) a personal website, and (3) a personal e-coach, who provides regular updates of the individual’s physical activity status by email and who gives advice to increase daily physical activities (Figure 1). By means of these elements, the program aims to increase awareness about one’s own physical activity behavior, to give feedback on recent actual physical activity, and to provide support to make sustainable changes in physical activity behavior.

The activity monitor of DirectLife is based on the Tracmor triaxial accelerometer and has been validated against doubly labeled water for the estimation of total 24-hour energy expenditure [16]. The DirectLife monitor is the consumer version of the Tracmor accelerometer. Intervention group participants received the program, including the accelerometer, directly after randomization at the first study visit. They then received a link by email for registration and access to the Web-based program. Participants were instructed to continuously wear the activity monitor throughout the day to measure daily physical activity. Data were uploaded through an Internet connection to the database of the commercial provider on a regular basis, ranging between daily and once per 14 days. After an initial 8-day “assessment period” starting 1 week after the study visit, in which the current level of daily physical activity was measured, a target was set by the DirectLife program to increase the level of daily physical activity during a 12-week Web-based interactive coaching program. Personalized targets were set by the DirectLife program and were defined as the absolute increase in physical activity compared to the individual’s baseline assessment data. For the whole group, this corresponded to a mean increase of approximately 10% in daily physical activity at week 12, increasing at a linear rate per week. All participants were given the option to decrease the personalized goal (within limits: increase of daily physical activity minimally to 5% instead of 10%) or to increase their personalized end goal (dependent on physical activity level of the last week).

Participants were given a target for daily physical activity, which increased weekly, and data from the accelerometer were used for daily feedback. Coaching included general recommendations on physical activities from real-life coaches, who were available for further questions and advice by email correspondence. For every participant, 1 of the e-coaches was available for the DirectLife program during the entire study period. These coaches were actual persons in contact with the participant through the intervention website or through email. The control group was placed on a 3-month waiting list after which they received access to the intervention program at the end of the study. No specific instructions regarding daily physical activity were given.

### Measurements

#### Baseline Questionnaire

Enrollment and follow-up took place from November 2011 to August 2012. In preparation of the first visit to the study center, all participants completed a Web-delivered questionnaire on education, smoking status, and medical history, including medication use. Education was categorized as low (primary education and lower vocational education), intermediate (secondary education and intermediate vocational education), or high (high vocational education and university).

#### Physical Activity Outcome

At baseline and 3-month follow-up, daily physical activity was measured for 7 days following the visit at the study center using a wrist-worn triaxial accelerometer (GeneActiv, Kimbolton, Cambs, UK). Wearing of the GeneActive monitors started on a random weekday depending on which weekday the participant was included for the first visit at the study center, and monitors were returned after 7 days by standard mail. We chose to assess the primary outcome using accelerometers other than the one included in the intervention program to avoid interpretation of the intervention as an outcome. The GeneActive monitors were worn 24 hours per day on the right wrist and ankle. The GeneActiv wrist accelerometer provides a simple summary statistic of total physical activity counts that has been validated for measuring daily physical activity against doubly labeled water [17]. As a derivative outcome, we calculated the minutes per day spent in moderate-to-vigorous intensity physical activity from the wrist accelerometer, which has been validated against indirect calorimetry [18]. Measurement frequency was set at 85.7 Hz and raw acceleration values (in *g*) were recorded continuously on each axis over 7 consecutive days. Further details on data processing can be found elsewhere [10]. Outcome assessment was done by an independent researcher who was blind to study arm allocation.

#### Other Outcomes

To calculate body mass index (BMI), body height was measured without shoes using a stadiometer. Body weight was assessed at both visits without shoes using a scale. Waist circumference was obtained in a standing position halfway between the anterior superior iliac spine and the lower rib. Hip circumference was measured halfway between the trochanter major and the iliac crest.

Lean body mass and body fat percentage were assessed by bioelectrical impedance analysis (Biostat 1500, Euromedix, Leuven, Belgium). Blood pressure was measured manually twice at each visit using a handheld sphygmomanometer after 5 minutes of lying down. The mean of the 2 consecutive measurements was used. Pulse rate was measured by hand at the wrist after at least 5 minutes of lying down. Grip strength was measured to the nearest kilogram 3 times using a Jamar handheld dynamometer (Sammons Preston, Inc, Bolingbrook, IL, USA) with the dominant hand. The highest value was used for analysis. Framingham risk scores were calculated using NIH criteria [19].

#### Metabolic Outcomes

Fasting blood samples were drawn from each participant at both visits in the morning. Samples were transferred to the laboratory within 2 hours, divided into single-use aliquots, and frozen at -80 °C. All serum measurements were performed in 1 batch after completion of the entire study with fully automated equipment. Fasting glucose, cholesterol, high-density lipoprotein (HDL) cholesterol, and triglyceride levels were determined using the Modular P2 analyzer (Roche, Almere, the Netherlands), fasting serum insulin using immunoassay by Immulite 2500 (DPC, Los Angeles, CA, USA). Glycated hemoglobin was determined by high performance liquid chromatography (Primus Ultra2, Trinity Biotech Company, Kansas City, MO, USA). C-reactive protein was determined using a high-sensitivity immunoassay (COBAS Integra, Roche, IN, USA). Low-density lipoprotein (LDL) cholesterol was calculated using the Friedewald formula in participants without hypertriglyceridemia [20].

#### End Point of the DirectLife Intervention

To assess the potential effects of the Web-based intervention, a subgroup was created from the intervention group including participants successful in reaching their individually targeted increase in daily physical activity indicated by the intervention program. In the last 3 weeks of the program, an average physical activity level per week was calculated and was compared to the personalized target of the corresponding week. A participant was defined as successful when the average of at least 2 of the 3 weeks reached their personalized target of the DirectLife program. It was noticed that a substantial number of participants reached the targeted personalized goals at the end of the 12-week program, but there was some variation in the last 3 weeks. Therefore, we defined “successful” as the participants who reached the target in at least 2 of the 3 last weeks of the program.

### Statistical Analyses

Baseline differences between the successful participants and the control group and between the entire intervention group and the control group were calculated using a *t* test for continuous data, a Mann-Whitney analysis for skewed data, and a chi-square test for categorical data. Differences between baseline and follow-up within groups were tested using a paired sample Student *t* test of the means. Differences between groups were calculated using linear regression and were adjusted for age and gender. All analyses were performed with SPSS version 20.0 (IBM, Armonk, NY, USA). Statistical significance was set at *P*<.05.

## Results

### Participant Characteristics

A detailed flow of recruitment and inclusion is outlined elsewhere [10]. In short, a total number of 631 individuals responded to the newspaper advertisement, of which 344 fulfilled the selection criteria. In total, 235 participants were randomized into the study: 119 in the intervention group and 116 in the control group. Of the 235 randomized participants, 226 (96.2%) completed the trial.

Of the 119 participants in the intervention group at baseline, 5 participants were lost to follow-up (4.2% of intervention group). Among the 114 participants who completed the trial, 50 participants (42.0% of intervention group) successfully reached their personalized physical activity target (“successful” participants). Of the 64 who were not defined as successful, 13 did not finish the DirectLife program (10.9% of intervention group) and 51 did not reach the personalized target (42.9% of intervention group).

Table 1 shows the baseline characteristics of the entire intervention group (n=119) and the successful participants (n=50) and both groups were compared with the entire control group (n=116). Male participants were more likely to successfully reach their personalized target for DirectLife compared to female participants. Of the successful participants, 26% (13/50) were female compared to 39.5% (47/119) in the entire intervention group. In-line with a difference in gender distribution, the average body height of the successful participants was higher compared to the control group. No other significant differences between groups were found.

On average, the personalized goals for males and females were similar. In the total intervention group, 36.1% (43/119) of participants changed their personalized goal: 15.1% (18/119) decreased the goal and 21.0% (25/119) increased the goal. There was no difference between men and women (*P*=.68). In the successful group, 44% (22/50) of the participants changed their personalized goal. There was an overrepresentation of participants increasing their goal (30%, 15/50) and men were more likely to increase their goal (38%, 14/37) compared to women (8%, 1/13; *P*=.038) (data not shown).

**Table 1:** Baseline characteristic of control group, total intervention group, and successful participants.^a^

Characteristics		Control group	Intervention group			
		Control group (n=116)	Intervention group (n=119)	*P* value for intervention vs control group	Successful participants (n=50)	*P* value for successful participants vs control group
**Demographics**						
	Sex (female), n (%)	49 (42.2)	47 (39.5)	.67	13 (26)	.047
	Age (years), mean (SD)	64.9 (2.8)	64.7 (3.0)	.61	64.6 (2.8)	.63
**Clinical parameters, mean (SD)**						
	Height (cm)	172.1 (9.3)	173.6 (9.9)	.25	175.7 (9.6)	.02
	Weight (kg)	86.3 (15.8)	87.4 (15.8)	.61	87.6 (15.6)	.64
	BMI (kg/m^2^)	29.1 (4.7)	28.9 (4.7)	.84	28.2 (3.7)	.25
	Waist circumference (cm)	101.4 (12.3)	102.3 (13.1)	.56	102.1 (12.2)	.74
	Fat percentage (%)	36.4 (8.1)	36.5 (7.6)	.95	34.5 (6.3)	.11
**Cardiovascular disease risk**						
	Framingham 10-year CVD risk (%), mean (SD)	11.3 (7.5)	11.9 (7.2)	.50	13.3 (7.5)	.10
**Physical activity**						
	5-day moderate-to-vigorous activity (min/day), median (IQR)	14.5 (8.2-32.5)	16.8 (7.8-26.4)	.43	20.0 (9.2-27.5)	.97
**Biochemistry**						
	Fasting venous glucose, mean (SD)	5.7 (0.8)	5.7 (0.7)	.94	5.6 (0.6)	.78
	Fasting insulin (mU/L), median (IQR)	10.8 (7.0-15.8)	11.5 (8.1-16.9)	.47	12.4 (8.0-19.8)	.32
	HbA_1c_ (%), mean (SD)	5.4 (0.3)	5.4 (0.3)	.44	5.4 (0.2)	.72
	HOMA index, median (IQR)	2.6 (1.7-4.3)	2.8 (2.0-4.3)	.48	3.0 (2.0-5.0)	.39
	Total cholesterol, mean (SD)	5.8 (1.0)	5.7 (1.1)	.74	5.6 (1.1)	.28
	HDL cholesterol, mean (SD)	1.4 (0.4)	1.5 (0.5)	.51	1.4 (0.5)	.93
	Triglycerides, median (IQR)	1.4 (1.1-2.0)	1.5 (1.1-2.0)	.65	1.5 (1.1-2.0)	.99
	LDL cholesterol, mean (SD)	3.6 (0.9)	3.6 (1.0)	.66	3.4 (1.0)	.25
	Total/HDL cholesterol ratio, mean (SD)	4.3 (1.3)	4.2 (1.3)	.65	4.2 (1.3)	.70
	C-reactive protein, median (IQR)	1.4 (0.8-4.1)	1.6 (0.8-3.1)	.83	1.7 (0.7-3.8)	.85

^a^ Data are presented as medians with interquartile range (IQR) when skewed. *P* values were calculated with *t* test (continuous data), Mann-Whitney (skewed data), or chi-square (categorical data).

### Effects on the Successful Participants

**Figure 2:**
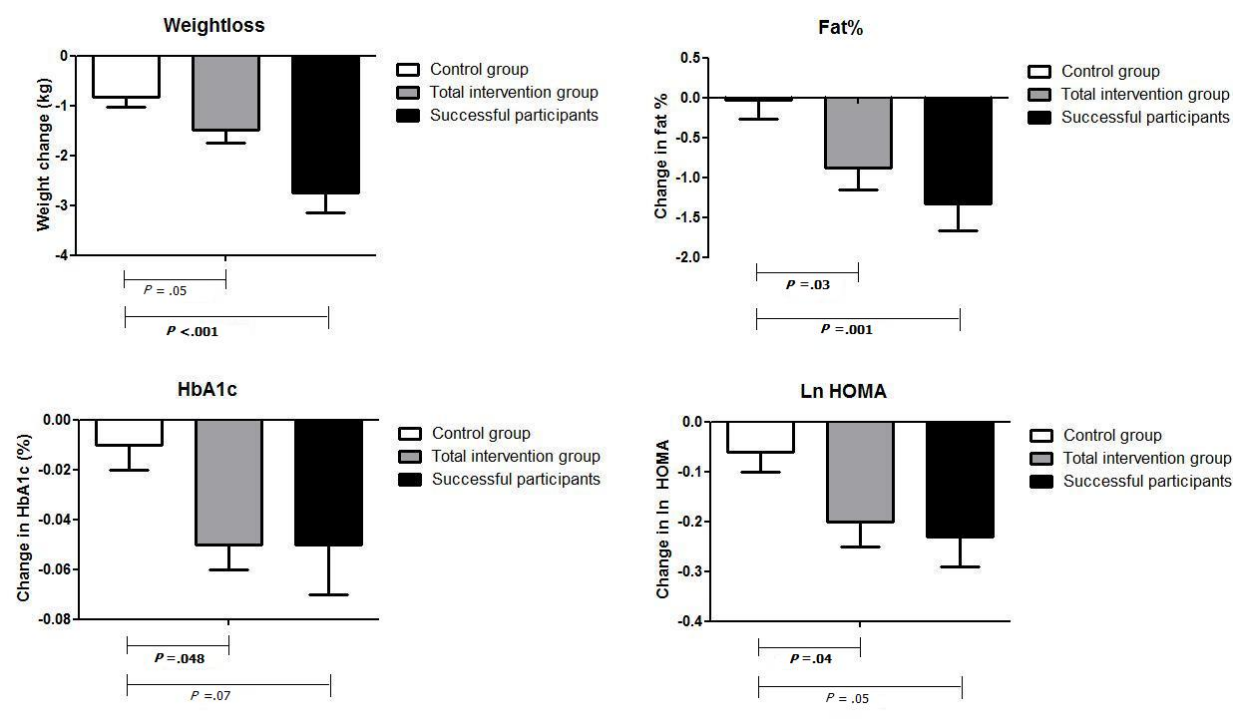
Effect of the intervention on selected parameters in the different study groups.

Table 2 and Figure 2 show the effects of the intervention at follow-up for the successful participants and the entire intervention group, both compared to the control group. Here we assess the magnitude of the effects in the group of successful participants and the total intervention group compared to the control group. Among the successful participants, time spent in moderate-to-vigorous intensity physical activity was higher (mean 18.8, SE 3.9 min/day) compared to the entire intervention group (mean 11.1, SE 2.1 min/day). The successful participants lost more body weight (mean 2.74, SE 0.40 kg) compared to the entire intervention group (mean 1.49, SE 0.26 kg), an 80% higher decrease. Beneficial effects were also seen for waist circumference with a decrease of mean 3.74 (SE 0.55) cm vs mean 2.33 (SE 0.36) cm in the successful participants vs the entire intervention group (62% higher decrease). In-line with the beneficial changes in body composition, significant improvements were seen in metabolic outcomes in the successful participants compared to the entire intervention group. Beneficial effects were seen for the homeostatic model assessment (HOMA) index, a marker for insulin resistance, with decreases of mean 0.23 (SE 0.06) and mean 0.20 (SE 0.05), respectively (15% higher decrease), and decreases for the cholesterol/HDL ratio of mean 0.39 (SE 0.11) and mean 0.20 (SE 0.07), a 95% higher decrease. For the Framingham risk score, a decrease was seen among the successful participants of 0.90% (SE 0.46) compared to 0.54% (SE 0.33) in the entire intervention group.

In a sensitivity analysis, we repeated all calculations with a different definition of “successful”; namely, only those who reached their personalized target in week 12 (n=40). We found similar results (data not shown).

**Table 2:** Results for clinical parameters and glucose metabolism of successful participants compared to the total control group.^a^

Characteristics		Control group (n=112)	Total intervention group (n=114)		Successful participants (n=50)		Relative increase^d^
		Mean Δ (SE)	Mean Δ (SE)	*P* ^b^	Mean Δ (SE)	*P* ^c^	
**Clinical parameters**							
	Weight (kg)	–0.82 (0.21)	–1.49 (0.26)	.05	–2.74 (0.40)	<.001	1.84
	BMI (kg/m^2^)	–0.29 (0.07)	–0.50 (0.09)	.07	–0.91 (0.13)	<.001	1.82
	Waist circumference (cm)	–1.29 (0.34)	–2.33 (0.36)	.04	–3.74 (0.55)	<.001	1.62
	Body fat (%)	–0.03 (0.24)	–0.88 (0.28)	.03	–1.33 (0.34)	.001	1.51
**Cardiovascular disease risk**							
	Framingham risk score (%)	–0.01 (0.31)	–0.54 (0.33)	.25	–0.90 (0.46)	.13	1.67
**Physical activity**							
	5-day moderate-to-vigorous activity (min/day)	–0.15 (1.5)	11.1 (2.1)	<.001	18.8 (3.86)	<.001	1.69
**Biochemistry**							
	Fasting venous glucose (mmol/L)	–0.13 (0.04)	–0.20 (0.05)	.32	–0.14 (0.06)	.77	0.70
	Ln^e^ insulin (mU/L)	–0.04 (0.04)	–0.16 (0.04)	.04	–0.20 (0.06)	.03	1.25
	HbA_1c_ (%)	–0.01 (0.01)	–0.05 (0.01)	.048	–0.05 (0.02)	.07	1.00
	Ln HOMA index	–0.06 (0.04)	–0.20 (0.05)	.04	–0.23 (0.06)	.05	1.15
	Total cholesterol (mmol/L)	–0.18 (0.05)	–0.25 (0.06)	.42	–0.38 (0.09)	.08	1.52
	HDL cholesterol (mmol/L)	–0.04 (0.02)	–0.008 (0.02)	.29	0.03 (0.03)	.07	N/A
	Ln^e^ triglycerides (mmol/L)	–0.06 (0.02)	–0.10 (0.03)	.35	–0.18 (0.04)	.02	1.80
	LDL cholesterol (mmol/L)	–0.11 (0.04)	–0.17 (0.04)	.43	–0.28 (0.07)	.08	1.65
	Cholesterol/HDL ratio	–0.05 (0.05)	–0.20 (0.07)	.12	–0.39 (0.11)	.007	1.95
	Ln^e^ C-reactive protein (mg/L)	–0.11 (0.09)	–0.12 (0.08)	.93	–0.24 (0.15)	.50	2.00

^a^ *P* values between groups were calculated with linear regression. All *P* values were adjusted for age and sex.

^b^ *P* value for total control group vs total intervention group.

^c^ *P* value for total control group vs successful participants.

^d^ Relative increase of successful participants compared to total intervention group.

^e^ Ln=natural logarithm. Natural logarithm presented when data at baseline skewed.

### Tertiles for Dose-Response Relationship

To explore the effect of physical activity, the entire intervention group was divided into tertiles based on the change in minutes spent in moderate-to-vigorous intensity physical activity. Because of technical errors, data on moderate-to-vigorous intensity physical activity counts were not available for 11 of 119 intervention group participants (9.2%), resulting in 3 tertiles with 36 participants. The lowest tertile showed, on average, a decrease of 6.75 (SD 8.20) minutes spent in moderate-to-vigorous intensity physical activity. The middle tertile showed on average an increase of 5.91 (SD 3.36) minutes and the highest tertile showed on average an increase of 34.37 (SD 21.52) minutes spent in moderate-to-vigorous intensity physical activity.

### Dose-Response Relationship of Physical Activity on Metabolic Outcomes

Finally, we assessed the association of the increase in physical activity levels with metabolic outcomes in a dose-response relationship. Table 3 shows the tertiles that were made based on increase in minutes spent in moderate-to-vigorous intensity physical activity as objectively measured with the wrist-worn accelerometer. At baseline, no differences were found between tertiles with regard to physical activity levels. When change in minutes spent in moderate-to-vigorous activity increased, more participants were defined successful (*P* for trend =.001). Furthermore, it was shown that there was a significant association of increasing physical activity with decreasing body weight (highest tertile: mean –2.85, SE 0.51 kg; lowest tertile: mean –0.93, SE 0.35 kg; *P* for trend=.004), decreasing BMI (highest tertile: mean –0.88, SE 0.17 kg/m^2^; lowest tertile: mean –0.34 SE 0.12 kg/m^2^; *P* for trend=.02), and reduction in waist circumference (highest tertile: mean –3.69, SE 0.72 cm; lowest tertile: mean –1.58, SE 0.52 cm; *P* for trend=.03). Furthermore, it was shown that with increasing physical activity, there was a higher improvement in Framingham risk score (highest tertile: mean –1.28%, SE 0.76%; lowest tertile: mean 0.43%, SE 0.46%; *P* for trend=.045). Also for metabolic outcomes, a dose-response was seen for (levels of) HDL cholesterol, cholesterol/HDL ratio, and triglycerides, but not for fasting glucose or HbA_1c_.

**Table 3:** Dose-response relationship of Δ minutes spent in moderate-to-vigorous physical activity with endpoints.^a^

Characteristics		Intervention group			*P* for trend
		Low (n=36)	Middle (n=36)	High (n=36)	
**Baseline moderate-to-vigorous activity**					
	Mean (SD)	22.5 (17.9)	17.3 (16.5)	19.0 (14.9)	.38
	Range	2.4 to 87.2	0.8 to 92.0	2.4 to 62.4	
**Δ minutes moderate-to-vigorous activity**					
	Mean (SD)	–6.75 (8.20)	5.91 (3.56)	34.27 (21.52)	
	Range	–25.8 to 1.60	1.70 to 14.2	15.8 to 117.6	
Successful participants, n		11	12	26	.001
**Clinical parameters (mean Δ, SE)**					
	Weight (kg)	–0.93 (0.35)	–0.64 (0.44)	–2.85 (0.51)	.004
	BMI (kg/m^2^)	–0.34 (0.12)	–0.23 (0.15)	–0.88 (0.17)	.02
	Waist circumference (cm)	–1.58 (0.52)	–1.92 (0.67)	–3.69 (0.72)	.03
	Fat percentage	–0.46 (0.48)	–0.44 (0.35)	–0.94 (0.43)	.43
**Cardiovascular disease risk (mean Δ, SE)**					
	Framingham risk score (%)	0.43 (0.46)	–0.78 (0.52)	–1.28 (0.76)	.045
**Biochemistry (mean Δ, SE)**					
	Glucose (mmol/L)	–0.23 (0.10)	–0.15 (0.09)	–0.21 (0.07)	.94
	Ln insulin (mU/L)	–0.14 (0.07)	–0.06 (0.08)	–0.26 (0.08)	.20
	HbA_1c_ (%)	–0.04 (0.02)	–0.03 (0.02)	–0.07 (0.02)	.20
	Ln HOMA index	–0.18 (0.08)	–0.09 (0.08)	–0.30 (0.08)	.26
	Total cholesterol (mmol/L)	–0.29 (0.08)	–0.10 (0.10)	–0.40 (0.11)	.63
	HDL cholesterol (mmol/L)	–0.10 (0.04)	0.01 (0.04)	0.05 (0.03)	.007
	Ln triglycerides (mmol/L)	0.003 (0.05)	–0.06 (0.05)	–0.22 (0.07)	.02
	LDL cholesterol (mmol/L)	–0.19 (0.06)	–0.09 (0.08)	–0.27 (0.09)	.61
	Cholesterol/HDL ratio	0.006 (0.08)	–0.15 (0.11)	–0.48 (0.17)	.02
	Ln C-reactive protein (mg/L)	–0.11 (0.10)	–0.17 (0.13)	–0.23 (0.17)	.66

^a^ *P* for trend was calculated with linear regression. *P* for trend was adjusted for sex and age, except for Framingham risk score for which age and sex are integrated.

## Discussion

### Principal Results and Comparison to Prior Work

The findings of the present study are fourfold. First, 42.0% (50/119) of the intervention group had reached their personalized target for daily physical activity. Second, we found almost doubled effects in body weight, BMI, and cholesterol/HDL ratio in those who successfully had reached their personalized daily physical activity target compared to the entire intervention group. Third, we found that men were more successful on reaching the personalized targets than women. Fourth, in the entire intervention group we found that metabolic outcome improved with increasing minutes spent on physical activity.

So far, studies have reported different results on improving daily physical activity in different age groups through a Web-based intervention. Limited studies report on elderly participants from the general population with regard to BMI, sedentary lifestyle, and comorbidities. Compared to a waiting list control group some studies reported an increase in moderate and vigorous intensity physical activity [21] or moderate intensity physical activity and walking [22], whereas other studies reported no significant differences in physical activity [23,24]. All these studies used self-report questionnaires for reporting on physical activity instead of objectively measured physical activity, making it hazardous to assess who had actually reached the targeted increase in physical activity. We report here that 42% of the entire intervention group had reached their personalized daily physical activity target when measured objectively. To our knowledge, data on participants successfully increasing objectively measured physical activity as targeted are very limited. The main reason for this is that very few of the studied interventions mention an objectively measured target increase. A randomized controlled trial performed among 1071 participants (mean age 53 years; 57% of participants with a BMI ≥25) studied the effect on nutrition and physical activity of a 12-week Internet program called Guide to Health (GTH only) that focused on nutrition and physical activity, the Guide to Health program plus a series of group-based support sessions (GTH+), and a waiting list control group. Physical activity was measured using a pedometer, counting steps/day with a target for all groups to increase physical activity with 2142 steps/day at posttreatment compared to baseline. In the GTH+ group, 41.7% increased the step count as targeted compared to the control group (24.4%, *P*=.07), showing that the GTH+ group tended to be more likely to reach the step goal. The GTH only group showed that 35.8% successfully increased their step count [25]. Although the physical activity in this study was objectively measured in an alternate way, our results show a comparable success rate.

Our study showed many beneficial effects on body composition and metabolic outcomes, with a significant average body weight loss of 2.74 kg in 3 months for the successful participants. Furthermore, our trial showed beneficial effects on body composition and metabolic outcomes also in the control group. The latter finding indicates that we have selected a motivated study population who, while on the waiting list for the intervention, may have adopted other strategies to improve their level of daily physical activity. Furthermore, the finding stresses the importance of a well-chosen control arm in clinical trials. Trials of other Web-based physical activity interventions on body composition showed various results [25-27]. For example, the aforementioned Guide to Health study comparing 2 Web-based interventions directed at healthy nutrition and physical activity reported small effects in randomly assigned individuals of -0.10 kg or -0.25 kg on body weight, none of them significant at long-term follow-up [25]. Other studies reporting the results of non-Web-based interventions directed at improving physical health in sedentary obese elderly people showed a mean body weight loss of 1.8 kg [28] or a mean body weight loss -3.6% [29]. In view of these results, our study showed a large effect on body weight. However, for studies primarily directed at body weight loss (including behavioral components other than physical activity), larger effects were seen compared to our study and most other studies directed at improving daily physical activity [30].

In the present study, we showed a general 1.2-fold to 2-fold larger effect in the successful participants compared to the entire intervention in most parameters. In the dose-response analysis, we observed that with tertiles of increasing objectively measured physical activity, there was an increase in beneficial effects of the intervention on body composition and metabolic outcomes. Of note, those with the lowest baseline physical activity level had the highest increase in physical activity level. The fact that there is a linear relationship between the increase in objectively measured physical activity and beneficial changes in body composition and metabolic outcomes indicates that there is also a beneficial effect of the intervention in participants who did not reach the targeted physical activity goal. An interpretation may be that intervention programs should focus on how to increase the compliance of participants to the program and to set feasible physical activity goals leading to higher physical activity levels in general for all participants.

Our analyses showed men were more likely to be successful compared to women; when successful, men were more likely to increase the personalized target compared to women. These are interesting findings. It is known that studies on the effects of physical activity interventions were mainly conducted in females, emphasizing the need for data about physical activity interventions targeted to men [31]. A possible explanation for our finding could be our recruitment strategy has unintentionally selected for more motivated men compared to the recruited women. This is also reflected in the fact that we recruited more males than females overall. Because we found the successful group to have a higher chance of increasing the target, this may reflect an overrepresentation of highly motivated participants in the successful group. In this group, men were more likely to increase their goal, suggesting that men were more motivated then women.

Additionally, it is likely that in this age group, men are more likely than women to already have adopted more digital and electronic gadgets to their lifestyle. For instance, in this age group it is likely that the men had more experience with working with computers than women because men in this age category were more likely to have a job with computers compared to women, especially because it was a highly educated group. Alternatively, using digital solutions and personalized targets may be more effective in men compared to women in contrast to working out in groups, for instance. This may be the case in our group of highly educated, physically inactive men. Future research is needed to confirm these speculations in this subgroup.

Although it was shown that men were more likely to successfully reach the targeted physical activity level compared to women, it was not the primary goal of the present paper to analyze the determinants of parameters that determine which factors predict which participants were successful in reaching their individualized physical activity target. Such a determinant analysis will be performed in forthcoming studies and will address different research questions with the ultimate aim to better target different populations.

Our analyses show a dose-response relationship between increasing physical activity on metabolic outcome. One consequence of this may be that reaching personalized targets is a useful way to stimulate individuals to adopt a lifestyle with a higher level of physical activity, but that the actual level of physical activity increase is less important. A flexible goal (as used in the present program) may, therefore, be very useful to keep participants motivated: even if the increase in physical activity is less than the initial target, there are still metabolic benefits

In the present paper, we stratified our main analyses into those who were successful versus those who were not and performed a dose-response analysis. We found that for those who were successful, the health benefits were larger than in the total intervention group. Furthermore, we report on gender as a determinant of success; men were more likely to increase their personalized goal, suggesting that men were more motivated. Although the intention-to-treat analysis of the total intervention group shows that the intervention is effective, our stratified analysis shows that there is a subgroup in whom the effects are much bigger and what characterizes these participants. This identifies whom to target with the current intervention, what other groups need an adjusted targeting, and what the effects of the intervention may be when applied optimally in the optimal population. All these lessons may help in the design and adjustment of the present and comparable interventions, especially in the targeted aging population

### Limitations

A drawback of our study is that we selected highly motivated participants who were able to use the Internet, leading to a population with a high education level. This hampers generalizability. Furthermore, men were more likely to reach the personalized physical activity goals successfully making the absolute results of the successful participants more difficult to interpret. Furthermore, we did not measure the food intake, which can be of great importance when studying the effect of the intervention. Food intake may have a direct effect on energy balance and several of the metabolic outcomes. Finally, in the present analyses we did not aim to investigate the determinants of who was going to be successful. Such analyses would, for instance, involve a determinant and cluster analyses and is the topic of ongoing analyses. A strength of this study is that we objectively measured physical activity. Furthermore, although participants were generally overweight, our study population consisted of volunteers in which comorbidities were present, increasing the generalizability toward the general elderly population. Furthermore, this study shows that men were more likely to successfully reach their personalized goals, which is a promising result for future interventions directed at improving physical activity. Furthermore, this study is unique in analyzing the dose-response relationship of physical activity within the intervention group leading to new insights for intervention programs. Finally, because the intervention is a Web-based intervention, it is likely to have a better cost benefit compared to face-to-face physical activity interventions.

### Conclusions

In conclusion, 42.0% (50/119) of the intervention group reached the end goal for daily physical activity, which was associated with a markedly better effect on metabolic outcomes compared to the effect in the entire intervention group. In this population, men were more successful at reaching the personalized physical activity targets. Findings demonstrate the large potential of Web-based interventions for improving health in the aging population by increasing physical activity, with possibilities for future improvements in increasing the proportion of the population reaching the targeted physical activity goal.

## Multimedia Appendix 1

CONSORT-EHEALTH checklist V1.6.2 [32].

## Abbreviations

AGO: Actief en Gezond Oud
BMI: body mass index
CVD: cardiovascular disease
GPPAQ: General Practice Physical Activity Questionnaire
HbA1c: glycated hemoglobin
HOMA: homeostatic model assessment
HDL: high-density lipoprotein
LDL: low-density lipoprotein
