# Supplementary material for: Dose-Response Effects of a Web-Based Physical Activity Program on Body Composition and Metabolic Health in Inactive Older Adults: Additional Analyses of a Randomized Controlled Trial
Source: J Med Internet Res. 2014 Dec 4;16(12):e265. doi: 10.2196/jmir.3643 (PMC4275504; doi:10.2196/jmir.3643)
Supplement: Supplementary file 1 [file jmir_v16i12e265_app1.pdf]

CONSORT-EHEALTH Checklist V1.6.2 Report

(based on CONSORT-EHEALTH V1.6), available at [http://tinyurl.com/consort-ehealth-v1-6].

2843

Date completed
7/23/2013 15:51:17
by
Carolien Wijsman

Effects of an Internet-based intervention on physical ativity and metabolism in older adults: a randomized controlled trial

TITLE

1a-i) Identify the mode of delivery in the title
web-based intervention'
1a-ii) Non-web-based components or important co-interventions in title
No important co-interventions were used.
1a-iii) Primary condition or target group in the title
in older adults'

ABSTRACT

1b-i) Key features/functionalities/components of the intervention and comparator in the METHODS section of the ABSTRACT
intervention group recieved Internet program Directlife, which was directed at increasing PA using monitoring and feedback by accelerometer and digital coaching'
1b-ii) Level of human involvement in the METHODS section of the ABSTRACT
No specific providers were involved in the program (not relevant).
1b-iii) Open vs. closed, web-based (self-assessment) vs. face-to-face assessments in the METHODS section of the ABSTRACT
" The primary outcome was relative increase in physical activity measured objectively using ankle- and wrist-worn accelerometers."
1b-iv) RESULTS section in abstract must contain use data
Two-hundred and twenty-six participants (97%) completed the study. At the ankle, activity counts increased by 46% (standard error (SE) 7%) in the intervention group, compared to 12% (SE 3%) in the control group (pdifference<0.001). Measured at the wrist, activity counts increased by 11% (SE 3%) in the intervention group and 5% (SE 2%) in the control group (pdifference = 0.11). After processing of the data, this corresponded to an increase of 11 minutes in moderate-to-vigorous activity in the intervention group versus 0 minutes in the control group (pdifference = 0.001). Weight decreased significantly more in the intervention group compared to controls (-1.5 kg vs. -0.8 kg respectively, p=0.046), as did waist circumference (-2.3 cm vs. -1.3 cm respectively, p=0.036) and fat mass (-0.6% vs. 0.07% respectively, p =0.025). Furthermore, insulin and Hba1c levels were significantly more reduced in the intervention group compared to controls (both p < 0.05).
1b-v) CONCLUSIONS/DISCUSSION in abstract for negative trials
" in inactive older adults, a 3-month web-based physical activity intervention was effective in increasing objectively measured daily physical activity and improving metabolic health"

INTRODUCTION

2a-i) Problem and the type of system/solution
" Intervention studies directed at increasing physical activity have been shown to be effective and may improve metabolism[8], also in older populations [9;10] . However, most of these interventions have used face-to-face communication, making them costly, time-consuming, thus hampering the potential of implementation as preventive programs at a larger scale.

Novel technologies, such as Internet and e-mail, nowadays provide interactive ways to administer digital coaching and feedback on physical activity, and have potential for wide-scaled implementation.

Moreover, it is unclear what the effect is of such web-based physical activity interventions on outcomes of metabolic health in old age"
2a-ii) Scientific background, rationale: What is known about the (type of) system
" A recent meta-analysis on web-based physical activity intervention studies showed promising results in increasing daily physical activity[11]. Many of these, however, used either small study populations, included study populations under the age of 60, or did not measure physical activity objectively. Moreover, it is unclear what the effect is of such web-based physical activity interventions on outcomes of metabolic health in old age[12;13] . "

METHODS

3a) CONSORT: Description of trial design (such as parallel, factorial) including allocation ratio
" In this randomized controlled trial, we examined whether a 3-month web-assisted intervention directed at increasing daily physical activity was effective in 60-70 year old inactive individuals. The intervention comprised an Internet program aimed at increasing physical activity using monitoring and feedback by accelerometer and digital coaching. Furthermore, we studied the effect of this intervention on metabolic health including anthropometric measures and markers of glucose and lipid metabolism."
3b) CONSORT: Important changes to methods after trial commencement (such as eligibility criteria), with reasons
Not relevant, as this did not occur. We used a fully developed program which is available for consumers in the market.
3b-i) Bug fixes, Downtimes, Content Changes
Not relevant, as this did not occur.
4a) CONSORT: Eligibility criteria for participants
" 1) age between 60 and 70 years, 2) no history of diabetes or use of glucose lowering medication, 3) absence of disability impeding increase in physical activity, 4) possession and use of personal computer with Internet connection. "
4a-i) Computer / Internet literacy
yes, see previous item
4a-ii) Open vs. closed, web-based vs. face-to-face assessments:
"The study recruited participants aged 60 to 70 years from the region of Leiden, The Netherlands. The recruitment strategy included advertisement in local newspapers and press notification, directing participants motivated to increase physical activity to the study website where they completed an online questionnaire."
4a-iii) Information giving during recruitment
The newspaper advertisement and the patient information were issued in Dutch , and are not included in the manuscript.
4b) CONSORT: Settings and locations where the data were collected

" At baseline and three-month follow up, daily physical activity was measured during seven days following the visit at the study center, using an ankle- and wrist worn tri-axial accelerometer (GeneActiv, Kimbolton, Cambs, UK). Wear was started on a random weekday, and GENEAs were returned after seven days by standard mail. "

4b-i) Report if outcomes were (self-)assessed through online questionnaires

Questionnaires were not used for primary outcomes, nor metabolic outcomes. Only questionnaires on baseline data: In preparation of the first visit to the study center, all participants completed an Web-delivered questionnaire on education, smoking status and medical history, including medication use.

4b-ii) Report how institutional affiliations are displayed

This is not specifically adressed in manuscript: the study was completely performed by hospital (LUMC), without before mentioning of program name

5) CONSORT: Describe the interventions for each group with sufficient details to allow replication, including how and when they were actually administered

5-i) Mention names, credential, affiliations of the developers, sponsors, and owners

" Subjects in the intervention group received a commercially available web-based physical activity program (Directlife, Philips, Consumer Lifestyle, Amsterdam) directed at increasing daily physical activity. "

5-ii) Describe the history/development process

not applicable, as the intervention was used in completed form.

5-iii) Revisions and updating

We used a fully developed non-dynamic intervention which is in the market for consumer use, therefore not applicable for this ms.

5-iv) Quality assurance methods

not applicable, not clear what is meant by ' information' in this sence. A fully developed, consumer availabe program was used.

5-v) Ensure replicability by publishing the source code, and/or providing screenshots/screen-capture video, and/or providing flowcharts of the algorithms used

Not applicable: the program is already in the market, and algorithms are not public.

5-vi) Digital preservation

Access is granted only after purchase (for non-study participants), intervention cannot be reached otherwise.

5-vii) Access

Intervention group participants received the program, including the accelerometer, directly after randomization at the first study visit. By e-mail they then received a link for registration and access to the web program.

5-viii) Mode of delivery, features/functionalities/components of the intervention and comparator, and the theoretical framework

" Intervention

Subjects in the intervention group received a commercially available web-based physical activity program (Directlife, Philips, Consumer Lifestyle, Amsterdam) directed at increasing daily physical activity. The DirectLife program is based on established health behavior change models[16;17], and takes into account the individual's current activity level, and subsequently provides a personal goal. Briefly, DirectLife consists of three elements: 1) an accelerometer-based activity monitor, 2) a personal website, and 3) a personal e-coach, who provides regular updates of the individual's physical activity status by e-mail and gives advice to increase physical activities. By means of these elements, the program aims to increase awareness about one's own physical activity behavior, to give feedback on recent actual physical activity, and to provide support to make sustainable changes in physical activity behavior. The activity monitor of DirectLife is based on the Tracmor tri-axial accelerometer, and has been validated against double labeled water for the estimation of total daily life energy expenditure[18]. The Directlife monitor is the consumer version of the Tracmor. Intervention group participants received the program, including the accelerometer, directly after randomization at the first study visit. By e-mail they then received a link for registration and access to the web program. Participants of the program were instructed to continuously wear the activity monitor throughout the day to measure daily physical activity. Data were uploaded through an Internet connection to the database of the commercial provider. After an initial eight-day "assessment period" starting one week after the study visit, in which the current level of daily activity was measured, a target was set to increase the level of daily activity during a twelve week web-based interactive coaching program. Participants were given a target for daily activity, which increased weekly, and data from the accelerometer were used for regular feedback. Coaching included general recommendations on physical activities and coaches were available for further questions and advice by e-mail correspondence.

The control group was placed on a three-month waiting list after which they received access to the intervention program, at the end of the study. No specific instructions regarding daily physical activity were given. "

5-ix) Describe use parameters

" After an initial eight-day "assessment period" starting one week after the study visit, in which the current level of daily activity was measured, a target was set to increase the level of daily activity during a twelve week web-based interactive coaching program. Participants were given a target for daily activity, which increased weekly, and data from the accelerometer were used for regular feedback. Coaching included general recommendations on physical activities and coaches were available for further questions and advice by e-mail correspondence.

The control group was placed on a three-month waiting list after which they received access to the intervention program, at the end of the study. No specific instructions regarding daily physical activity were given. "

5-x) Clarify the level of human involvement

" Coaching included general recommendations on physical activities and coaches were available for further questions and advice by e-mail correspondence. All participants were in contact with one of the digital coaches available for the Directlife program during the entire study period."

5-xi) Report any prompts/reminders used

The prompts were set by the intervention program, and differed per individual. We do not own this algorhytm, and information is not in the ms.

5-xii) Describe any co-interventions (incl. training/support)

no co-interventions were used.

6a) CONSORT: Completely defined pre-specified primary and secondary outcome measures, including how and when they were assessed

" At baseline and three-month follow up, daily physical activity was measured during seven days following the visit at the study center, using an ankle- and wrist worn tri-axial accelerometer (GeneActiv, Kimbolton, Cambs, UK). Wear was started on a random weekday, and GENEAs were returned after seven days by standard mail. We chose to assess the primary outcome using accelerometers other than the one included in the intervention program to avoid interpretation of the intervention as an outcome. Both GeneActive monitors were worn 24-hours per day on the right side. The GeneActive wrist accelerometer provides a simple summary statistic of total activity counts has been validated for measuring daily physical activity against doubly-labeled water[19] . We chose to additionally assess total activity counts using an ankle accelerometer as we hypothesized that this location would be more sensitive to walking and cycling behavior[20;21], the latter being a very frequent activity in our target population in The Netherlands. Primary outcome was the individual's relative change in activity counts after the intervention compared to baseline, measured at wrist and ankle. As a derivative outcome, we calculated from the wrist accelerometer the minutes per day spent in moderate or vigorous activity, which has been validated against indirect calorimetry[22].
Measurement frequency was set at 85.7 Hz and raw acceleration values in "g" were recorded continuously on each axis over seven consecutive days. Prior to processing, data were plotted for visual identification of non-wear and device faults. Non-wear was determined visually using thresholds of movement in combination with self-reported non-wear from participants. Short periods of non-wear (e.g. bathing) were accepted, and data for these periods were not imputed. Accelerometer data from participants contributing geneActiv data for five days or more within the seven day period were included in the analysis. Data from each axis were processed by a high pass RC filter (fc = 0.27 HZ) before computation of the resultant acceleration (R= ((x2 + y2 + z2)0.5) for each recorded time point. The average of each 24-hour integral of these values over the first five days was used as the average daily activity count for the assessment period. As measure for physical activity we used total activity counts recorded at both the ankle and the wrist accelerometer independently. Data collected from the right wrist of each participant over five days of continuous movement monitoring were processed to yield activity counts for successive one minute epochs and classified according to the appropriate MET cut-off points according to the method of Esliger[22], to establish the average number of minutes (epochs) daily spent in moderate and vigorous physical activity. Outcome assessment was done by an independent researcher who was blind to study arm allocation (MC).

Secondary outcomes
Body height was measured without shoes using a stadiometer. Body weight was assessed at both visits without shoes using a scale. Waist circumference was obtained in a standing position halfway between the anterior superior iliac spine and the lower rib. Hip circumference was measured halfway between the trochanter major and the iliac crest.
Lean body mass and body fat percentage were assessed by bio-electrical impedance (BIA) analysis (Biostat 1500, Euromedix, Leuven, Belgium). Blood pressure was measured twice at each visit using a hand-held sphygmomanometer after five minutes of lying down. The mean of the two consecutive measurements was used. Heart rate was measured at the wrist after at least five minutes of lying down. Grip strength was measured to the nearest kilogram three times using a Jamar hand dynamometer (Sammons Preston, Inc., Bolingbrook, IL, USA) with the dominant hand. The highest value was used for analysis. Framingham risk scores were calculated using NIH criteria [23]. "

6a-i) Online questionnaires: describe if they were validated for online use and apply CHERRIES items to describe how the questionnaires were designed/deployed

Questionnaires were not used for outcomes in this manuscript.

6a-ii) Describe whether and how "use" (including intensity of use/dosage) was defined/measured/monitored

" For per-protocol analysis, we included in the intervention group only those participants who finished the 12-week plan of the intervention program. All analyses were performed with SPSS version 20.0 (IBM, Armonk, NY, USA). Statistical significance was accepted at P < 0.05. "

6a-iii) Describe whether, how, and when qualitative feedback from participants was obtained

Not applicable for primary/secondary outcomes). Focus group was performed after study ended, and is not in this manuscript.

6b) CONSORT: Any changes to trial outcomes after the trial commenced, with reasons

There were no changes in trial outcomes.

7a) CONSORT: How sample size was determined

7a-i) Describe whether and how expected attrition was taken into account when calculating the sample size

" Sample size calculation
The sample size of the study was based on the assumption of a mean 10% higher increase (standard deviation 25%) in daily physical activity counts as measured using accelerometers on the ankle and wrist in the intervention group compared to the control group during a 3-month period. For this effect size with a power of 0.80 at alpha 0.05 (two sided), we calculated a sample size of 198 participants for the intention-to-treat analysis. Based on an estimated drop-out rate of 15%, we aimed to include 232 participants, and stopped after successful inclusion of 235 participants. "

7b) CONSORT: When applicable, explanation of any interim analyses and stopping guidelines

As we did not expected or studies adverse outcomes, we did not include guidelines in this manuscript.

8a) CONSORT: Method used to generate the random allocation sequence

Not applicable.

8b) CONSORT: Type of randomisation; details of any restriction (such as blocking and block size)

" At the baseline visit subjects were randomly assigned to the intervention group or a waitlist control group by the study physician or research nurse. Randomization was performed by a computerized program for intervention versus waitlist control in a ratio of 1:1, with a block size of 12. Stratification was performed by sex. Concealment of treatment allocation was ensured by randomizing at the end of the first study visit, after all baseline measurements and instructions at the study center were completed. "

9) CONSORT: Mechanism used to implement the random allocation sequence (such as sequentially numbered containers), describing any steps taken to conceal the sequence until interventions were assigned

" Concealment of treatment allocation was ensured by randomizing at the end of the first study visit, after all baseline measurements and instructions at the study center were completed. "

10) CONSORT: Who generated the random allocation sequence, who enrolled participants, and who assigned participants to interventions

" Randomization was performed by a computerized program for intervention versus waitlist control in a ratio of 1:1"

"At the baseline visit subjects were randomly assigned to the intervention group or a waitlist control group by the study physician or research nurse. "

11a) CONSORT: Blinding - If done, who was blinded after assignment to interventions (for example, participants, care providers, those assessing outcomes) and how

11a-i) Specify who was blinded, and who wasn't

" Blinding of this intervention was not possible and therefore not applied. "

11a-ii) Discuss e.g., whether participants knew which intervention was the "intervention of interest" and which one was the "comparator"

" The control group was placed on a three-month waiting list after which they received access to the intervention program, at the end of the study. No specific instructions regarding daily physical activity were given. "

11b) CONSORT: If relevant, description of the similarity of interventions

Not relevant.

12a) CONSORT: Statistical methods used to compare groups for primary and secondary outcomes

" Statistical analyses
Differences between baseline and follow up within groups were tested using a paired sample Student t test of the means. For skewed variables, In transformation was used. The effect of the intervention on physical activity was assessed by an unpaired 2-sided t test, comparing the relative change in daily physical activity counts between the intervention group and control group. For relative change in moderate-to-vigorous physical activity, a non-parametric test was used due to skewness of data. The effect of the intervention on secondary outcomes was assessed using an unpaired 2-sided t test, comparing the change in the secondary outcome between the intervention group and control group. Primary analyses were performed by intention-to-treat principle. Our study did have one follow-up measurement only, and loss to follow-up was very low. We therefore did not use imputation to replace our data, and participants of whom data was lost were not in the ITT analysis. For per-protocol analysis, we included in the intervention group only those participants who finished the 12-week plan of the intervention program. All analyses were performed with SPSS version 20.0 (IBM, Armonk, NY, USA). Statistical significance was accepted at P < 0.05. "

12a-i) Imputation techniques to deal with attrition / missing values

" Primary analyses were performed by intention-to-treat principle. Our study did have one follow-up measurement only, and loss to follow-up was very low. We therefore did not use imputation to replace our data, and participants of whom data was lost were not in the ITT analysis. "

12b) CONSORT: Methods for additional analyses, such as subgroup analyses and adjusted analyses

" For per-protocol analysis, we included in the intervention group only those participants who finished the 12-week plan of the intervention program. "

RESULTS

13a) CONSORT: For each group, the numbers of participants who were randomly assigned, received intended treatment, and were analysed for the primary outcome

"Figure 1 shows the inclusion flow chart of participants. A total of 631 participants completed the questionnaire on the study website. Of those, 235 participants (37%) met inclusion criteria and were randomized: 119 in the intervention arm, 116 in the control arm. Nine participants did not complete the study; five and four participants in the intervention and control group, respectively. Final analyses of outcomes therefore included 114 participants in the intervention group and 112 control participants. "

13b) CONSORT: For each group, losses and exclusions after randomisation, together with reasons

Is shown in CONSORT flow diagram (figure 1).

13b-i) Attrition diagram

"Adherence to the intervention program
All 114 participants who completed the study in the intervention arm received the intervention program during the visit to the study site at baseline. Of these, 109 participants (95.6%) started the intervention program after completing the first assessment week of the intervention program. In total, 104 participants (91.2%) completed the 12-week intervention program."

14a) CONSORT: Dates defining the periods of recruitment and follow-up

"Measurements
Enrollment and follow-up took place from November 2011 to August 2012."

14a-i) Indicate if critical "secular events" fell into the study period

Not applicable

14b) CONSORT: Why the trial ended or was stopped (early)

Not applicable

15) CONSORT: A table showing baseline demographic and clinical characteristics for each group

Is shown in table 1 of the manuscript.

15-i) Report demographics associated with digital divide issues

" Baseline characteristics of randomized participants are shown in table 1. The study groups were similar for all parameters. The majority of participants were male, and middle or highly educated."

16a) CONSORT: For each group, number of participants (denominator) included in each analysis and whether the analysis was by original assigned groups

16-i) Report multiple "denominators" and provide definitions

"Accelerometer data were available for 107 intervention and 109 control participants for tankle monitors, and 108 and 105 intervention and control partipants for wrist monitors, respectively. "
All data were available for secondary outcome analyses, as shown in table 2.

16-ii) Primary analysis should be intent-to-treat

" Primary analyses were performed by intention-to-treat principle."

17a) CONSORT: For each primary and secondary outcome, results for each group, and the estimated effect size and its precision (such as 95% confidence interval)

" Primary outcome: physical activity
Accelerometer data were available for 107 intervention and 109 control participants for tankle monitors, and 108 and 105 intervention and control partipants for wrist monitors, respectively. After 13 weeks, daily physical activity as measured by the ankle accelerometer increased by 46% (SE 7%, p < 0.001) in the intervention group, compared to 12 % (SE 3%, <0.001) in the control group (p for difference <0.001). Daily physical activity measured by the wrist accelerometer increased by 11% (SE 3%, p <0.001) in the intervention group, and by 5% (SE 2%, p = 0.027) in the control group (p for difference = 0.11). In the intervention group, there was a mean increase of 11.1 minutes per day (SE 2.1) spent in moderate-to-vigorous activity, compared to a mean decrease of 0.1 minutes (SE 1.5) in the control group (p for relative difference = 0.001) (Figure 2). In the per-protocol analysis, taking into account only those 91% (n=104) of participants who completed the intervention phase of the DirectLife program, results did not change. "
For secondary outcomes, all effect sizes and precision are shown in table 2.

17a-i) Presentation of process outcomes such as metrics of use and intensity of use

Continuous outcomes of exposure other than the before mentioned attrition rates were not used in this study.

17b) CONSORT: For binary outcomes, presentation of both absolute and relative effect sizes is recommended

No binary outcomes were used.

18) CONSORT: Results of any other analyses performed, including subgroup analyses and adjusted analyses, distinguishing pre-specified from exploratory

"In the per-protocol analysis, taking into account only those 91% (n=104) of participants who completed the intervention phase of the DirectLife program, results did not change. " For primary outcomes and:
" In the per-protocol analysis, taking into account only those 91% of participants who completed the intervention phase, differences did not materially change." for secondary outcomes

Other subanalyses were not performed in this study .

18-i) Subgroup analysis of comparing only users

"In the per-protocol analysis, taking into account only those 91% (n=104) of participants who completed the intervention phase of the DirectLife program, results did not change. " For primary outcomes and:
" In the per-protocol analysis, taking into account only those 91% of participants who completed the intervention phase, differences did not materially change." for secondary outcomes

Other subanalyses were not performed in this study .

19) CONSORT: All important harms or unintended effects in each group

No harms or side effects were expected or reported.

19-i) Include privacy breaches, technical problems

This did not occur.

19-ii) Include qualitative feedback from participants or observations from staff/researchers

This was not subject of study in our trial, but is subject of the focus group, which was performed after the study ended. This is therefore not mentioned in this manuscript.

DISCUSSION

20) CONSORT: Trial limitations, addressing sources of potential bias, imprecision, multiplicity of analyses

20-i) Typical limitations in ehealth trials

The study used blinded outcomes (accelerometers) instead of self-reporting. This is discussed: " The main strength of our study was the use of objectively measured daily physical activity. The majority of studies directed at increasing physical activity used self-reported physical activity measures, which could have resulted in an overestimation of the effect size. More recent studies used pedometers, which are unable to assess all types of physical activity and to give direct feedback to the wearer. With the use of tri-axial accelerometers, outcome assessment was blinded for participants as well as for study physicians and nurses. "
Also, attrition rates were very high.

21) CONSORT: Generalisability (external validity, applicability) of the trial findings

21-i) Generalizability to other populations

"Our study population consisted of highly educated and motivated participants, which may hamper the generalizability of our results. Future study should assess the effects of web-based interventions in elderly in a primary care setting using a population that better represents the general population. "

21-ii) Discuss if there were elements in the RCT that would be different in a routine application setting

Not applicable, as intervention was similar to routine application.

22) CONSORT: Interpretation consistent with results, balancing benefits and harms, and considering other relevant evidence

22-i) Restate study questions and summarize the answers suggested by the data, starting with primary outcomes and process outcomes (use)

"In this study, we describe the effect of a web-assisted physical activity intervention. We found a significant increase in daily physical activity in the intervention group compared to the control group. Furthermore, we showed that the intervention resulted in a significant improvement in body composition and parameters of glucose metabolism compared to the control group."

22-ii) Highlight unanswered new questions, suggest future research

" Furthermore, there is a need for better understanding the measurements of physical activity patterns from the accelerometer to further underpin the behavior changes associated with such interventions."

" However, it is unclear whether compliance can be sustained and whether long-term positive effects can be expected. The very few studies that reported on longer term follow-up and showed a significant increase in PA also after a shorter follow-up period, suggested that physical activity may increase further after 12 compared to 6 months[31] . This study, however, was performed in a primary care setting and used non-web-based digital intervention methods such as face-to-face counseling. Evidence for long-term effectiveness of web interventions is therefore required."

" Future study should assess the effects of web-based interventions in elderly in a primary care setting using a population that better represents the general population. A drawback of the present study was that did not record any dietary behaviors. It is possible that changes in diet account for a proportion of the observed beneficial effects on metabolic health. On the one hand, it would be interesting to study which dietary changes associated with increasing metabolic health and insight in such behavior could increase the potential to increase effectiveness of such the intervention by specific coaching on this subject. On the other hand, a potential implicit role for dietary factors in the observed effect in the present study does not mitigate the relevance of these results. Another drawback is the fact that we did not have data on longer term follow-up."

Other information

23) CONSORT: Registration number and name of trial registry

Dutch Trial Registry (www.trialregister.nl), NTR 3045

24) CONSORT: Where the full trial protocol can be accessed, if available

"Dutch Trial Registry (www.trialregister.nl), NTR 3045"

25) CONSORT: Sources of funding and other support (such as supply of drugs), role of funders

"Funding
This project was financially supported by Philips Consumer Lifestyle, and the Netherlands Genomics Initiative/Netherlands Organization for scientific research (NGI/NWO; 05040202 and 050-060-810). The funders had no role in the design and performance of the study, nor in the analyses or interpretation of the data or in the drafting of the manuscript. CAW and SPM take full responsibility for the integrity of the data. The authors report no conflict of interest. "

X26-i) Comment on ethics committee approval

" Written informed consent was obtained from all subjects. The study was approved by the medical ethical committee of Leiden University Medical Center, The Netherlands. "

x26-ii) Outline informed consent procedures

" Written informed consent was obtained from all subjects."

X26-iii) Safety and security procedures

"An independent physician was available for questions regarding study information. "

X27-i) State the relation of the study team towards the system being evaluated

"The authors report no conflict of interest. "
